# mHealth Apps for Enhanced Management of Spinal Surgery Patients: A Review

**DOI:** 10.3389/fsurg.2020.573398

**Published:** 2020-10-23

**Authors:** Michael Y. Bai, Ralph J. Mobbs, William R. Walsh, Callum Betteridge

**Affiliations:** ^1^NeuroSpine Surgery Research Group (NSURG), Sydney, NSW, Australia; ^2^Neuro Spine Clinic, Prince of Wales Private Hospital, Randwick, NSW, Australia; ^3^Faculty of Medicine, University of New South Wales (UNSW), Sydney, NSW, Australia; ^4^Wearables and Gait Assessment Research (WAGAR) Group, Prince of Wales Private Hospital, Randwick, NSW, Australia; ^5^Surgical Orthopedic Research Labs (SORL), University of New South Wales (UNSW), Sydney, NSW, Australia

**Keywords:** surgery, spine, mHealth, smartphone, apps

## Abstract

mHealth (mobile health) refers to mobile technologies that aid medical and public health practices. As of February 2019, 81% of Americans own a smartphone, and mHealth applications (apps) have become increasingly common with more than 400,000 mHealth applications currently available. Advancements in mobile technology now allow us to provide personalized up-to-date information, track personal health data, remind and engage patients, and communicate in a cost-effective way. There are new opportunities for healthcare providers to integrate mHealth into clinical practice. We discuss the current scientific evidence, and research into mHealth technology.

## Introduction

mHealth (mobile health) refers to mobile technologies that aid medical and public health practices (1). As of February 2019, 81% of Americans own a smartphone (2), and mHealth applications (apps) have become increasingly common with over 400,000 mHealth applications currently available to download on the Google Play Store and Apple App Store (3). Advancements in mobile technology allow us to provide personalized up-to-date information, track personal health data, remind and engage patients, and communicate in a cost-effective way. This has created new opportunities for healthcare providers to integrate mHealth into clinical practice. However, most mHealth apps are not based on scientific evidence, and there are calls for further research to enhance the scope and utilization of mHealth technology (4).

Post-operative communication with patients is a desirable part of safe and effective clinical care. However, effective pathways for communication may be difficult and may result in patients making unplanned and potentially unnecessary visits to their general practitioner or local emergency department. They may also take up more resources in terms of time spent on screening phone calls and emails by administrative staff (5). The use of smartphone technology provides secure methods to link patients directly with care providers to streamline the patient experience and optimize the use of limited resources.

The use of mHealth in neurosurgery is becoming a hot topic, with many researchers from around the world publishing their successes regarding the use of apps with spinal surgery patients. Spinal surgery patients face important challenges in post-op rehabilitation, pain management, and complications. Any advancement in a positive direction has the benefit of increasing quality of life and patient satisfaction. A survey conducted in 2017 has shown that three-quarters of spinal surgery patients would be interested in using a mHealth app to track their post-operative progress and communicate with their care team (6).

The aim of this review is to summarize the current literature regarding mHealth in the field of spinal surgery and discuss the benefits of this useful technology.

## Review

### Enhanced Information Delivery

Smartphone technology can be used to solve the major issues of inconsistent information, difficulty accessing information, and delayed communication, which are faced by many surgical patients (7). Compared to large, generic, printed patient information booklets, mHealth apps make it easier for patients to find important information related to their surgery and provide them with a convenient way to revisit personalized information provided to them at their clinic visit. Studies have shown that patients have poor retention of medical information, and an app can improve the patients understanding of their diagnosis, proposed surgery, required investigations, and changes they need to make to their medications (8). mHealth apps also help remind patients of crucial tasks they must complete in preparation for their surgery, such as stopping anticoagulants (7).

The Amie™ app by FavorHealth was studied by Stewart et al., who showed that preventable surgical cancellations due to poor compliance with pre-operative instructions could be reduced by using personalized push notifications to a group of neurosurgical patients (8). Push notifications also help remind patients to follow pre-operative instructions on the correct days. An app that requires patients to acknowledge notifications can also relay information back to care providers and give them a chance to contact the patient directly to remind them of pre-operative instructions if necessary (9).

Patients are also more likely to adhere to post-operative and discharge instructions when using mHealth apps (10). Those who engage with mHealth apps show greater patient satisfaction, better medication adherence, improved clinic attendance, lower readmission, and emergency department visits post-operatively (11). One study found that in the absence of an mHealth application, up to 55% of patients would have returned to the hospital for assessment of their wounds (12).

### Behavior and Activity Modification

The proof of concept for mHealth apps has been demonstrated in many other specialties, and these give insight into what makes mHealth apps successful and worthwhile. mHealth apps have been successful in behavior modification for secondary prevention of cardiovascular disease (13), substance misuse (14), and medication compliance (15). It is clear that interventions for behaviors such as physical activity, smoking, and opioid misuse would benefit patients undergoing spinal surgery. The experience of other mHealth apps can help inform the development of an application made specifically for spinal surgery patients, which can be as simple as scheduled reminders to complete a symptomatic questionnaire. Encouraging these behaviors adds diagnostic value for conditions that are partially diagnosed by symptom profile, like Lumbar Spinal Stenosis (16), and provides a longitudinal view of the symptom progression for clinical assessment of disease status.

Penn Medicine's NeuroPath app is one such mHealth app that has been piloted in spinal surgery patients (17). This app utilizes principles from enhanced recovery after surgery (ERAS) to encourage patients to do daily tasks, provide instructions and education, allow them to track and share their activity levels and medication usage and communicate directly with their care team (17). This app presented patients with daily to-do lists that included specific instructions depending on the time since their surgery, such as exercise, diet, wound care instructions, and requested patients to input their symptoms daily. Care providers can then interact with patients and provide feedback based on the information they uploaded to the app.

Postural and walking stability is an important objective gait metric for patients with spinal pathology. Changes in posture and walking stability, including falls, correlates with recovery following any spinal intervention. Yoong and authors noted, however, that mHealth apps and wearable devices to assist with the monitoring of relevant metrics following spinal interventions is still in its infancy with further device and app development to be done (18).

### Improved Follow-Up

mHealth apps offer a cost-effective way to safely allow earlier discharge while maintaining close follow-up with patients post-operatively (19, 20). It can save time for care providers and patients and reduce unnecessary transfers. A French study which looked at the use of an mHealth app for the post-operative follow up of patients who underwent outpatient microdiscectomy found that 94% of issues could be handled remotely. This allows the patient to have direct access to their care team and avoid unnecessary visits to their primary care physician or return to the hospital (21). The costs associated with implementing the app and maintaining a team to respond to alerts can be offset by a reduction in length of hospital stay (22).

There is also the potential that certain patients who communicate good progress via their mHealth app may not need to return for an in-person follow-up appointment. This could reduce the costs associated with running clinics and allow health care providers to focus their resources on other activities (7).

### Improved Outcomes

mHealth is an extremely promising modality for improving patient care and outcomes. It has been shown to improve patient experiences by involving patients in their own management which provides more confidence to patients after they have been discharged. Patients engage more with mHealth apps that have personalized content, a higher frequency of text messages, and two-way communication (13).

mHealth apps are extremely useful in the post-operative period for wound monitoring. Surgical site infections, if recognized early, can be safely managed as an outpatient, whereas delays in recognition can lead to readmission, patient stress, and an increased burden on the healthcare system (7). The technology exists to allow secure transfer of photographs of surgical wounds from patients to care providers via an encrypted smartphone app. This solution would decrease complication rates and increase patient satisfaction.

A randomized controlled trial of patients in China post lumbar spinal surgery explored the use of an mHealth app to deliver rehabilitation (23). The app allowed patients to view personalized rehabilitation plans made by their physicians and provided daily reports and prompts to encourage continued use of the app. Patients could also communicate with their doctors through the app, and they could make changes to their rehabilitation plans. This resulted in a significant improvement in their disability index and pain scores after 2 years compared to traditional rehabilitation (23). Their study also demonstrated increased benefits with higher app compliance.

### Increased Patient Satisfaction

A systematic review of the ability of smartphone apps to enhance communication with surgical patients was published by De La Cruz Monroy et al. in 2019 (7). They noted an overwhelmingly positive response from patients and care providers who are using mHealth apps in the perioperative setting.

Patients are willing to engage with mHealth apps and use them daily as they feel they are personal to their care. They also provide a sense of security as there is a continuous link to their care providers, and give the patients the opportunity to be involved in research. Some even report using the app simply because they were bored (24). mHealth apps have the potential to provide continuous 24-h monitoring for patients without overburdening the healthcare system. They also have the potential to increase patient compliance by featuring individualized feedback and rewards to patients.

The mHealth app “e-fitback” (Nouveal, e-santé) was used in a study of ERAS for patients undergoing spinal fusion for degenerative conditions (22). It collected post-operative information on pain, temperature, voiding, motor disorders, and blood-stained dressings. The app then triggered an alarm that would instigate a phone call from the ERAS team if needed. Patients responded well to the app, with 82.3% of users reporting feeling satisfied or very satisfied with the mHealth app as a tool to optimize patient care.

### Future Directions

One of the fears regarding the utilization of mHealth is that elderly patients would not engage with the technology. A pilot study by von Glinski et al. has demonstrated that as long as patients own and use a smartphone, mHealth apps are well-received by elective spinal surgery patients regardless of age, gender, or procedure invasiveness (5). This is promising and encourages the future development of more apps tailored to surgical patients. Another barrier to the implementation of mHealth apps is privacy concerns. Privacy concerns may relate to the amount of information that the clinician has access to, or the potential for hacking and data leaks which may result in the patient's health data being publicly available. When filling in a questionnaire regarding data privacy using mHealth apps, patients in lower socioeconomic classes had fewer concerns regarding privacy and were more likely to use an mHealth app, meaning greater benefit amongst (25). However, Limited uptake by wealthier individuals diminishes the overall benefit of widespread implementation of mHealth applications in health. Finally, the 2014 annual study on mHealth application development (26) predicted two further barriers to mHealth application uptake over the 2014–2019 period, practitioner resistance and difficult discoverability of individual applications due to the sheer number of applications available.

mHealth apps may be beneficial for spinal surgery patients with poor access to rehabilitation services (23) or may be useful in complementing traditional services. By reducing the burden of traditional services, it may free up resources so that more patients may benefit without compromising the quality of the service. There is also further potential for mHealth apps to integrate with add-on devices to enhance post-operative monitoring. These could include simple activity monitors or specialized medical devices designed for specific purposes (7). Such communication between a smartphone and external device has already demonstrated clinical utility in spinal surgery (27). Additionally, the processing power of handheld phones could allow mHealth apps to extrapolate the unprocessed data from external devices to generate a much more complex picture than what the device itself could provide. For example, it is possible to use the accelerometry data captured by a smartwatch to calculate gait velocity, smoothness, or asymmetry, rather than just step count.

Integration of data from a mHealth app to a patient's electronic health record (EHR) (28) would allow the data from the mHealth app to be stored on a centralized database for the hospital or local health district (28). This means that while the data would primarily be used by the spinal surgeon and the patient, other clinicians who adopt care of the patient can access the information as well, allowing for better integration between primary, secondary and tertiary care systems.

As it stands, the data captured by mHealth apps is limited to questionnaires and simple measures of health like heart rate and step count by the storage and processing capacity of handheld devices. With advancements in mobile phone technology, it stands to reason that the phone would be able to obtain more complex measures of health. For example, improvements in the sampling rate of the phone's internal accelerometers and gyroscopes, and an increase in phone processing power may allow for constant measurement of gait velocity and smoothness, which could be useful for an mHealth app specific to spinal surgery patients. As the amount of health data captured per minute increases, so does the need for a system capable of sorting through this data, and deciding what is useful or important for the clinician to know, as does the need to quickly upload this data. Thus, some adjuvant technologies such as artificial intelligence (AI) and cellular networks with greater bandwidth (5G+) may play a role in this future when mHealth apps and handheld phone technology are optimized.

## Conclusion

In conclusion, there have been several mHealth apps specially designed for and tested with spinal surgery patients reported in literature worldwide. Early experiences have shown that mHealth apps are a cost-effective way to provide safe, beneficial, and satisfactory care to patients. mHealth apps enhance two-way communication between patients and their care providers. They help to deliver patient information in a convenient and individualized fashion. Rehabilitation and follow-up care can be optimized via mobile apps also, allowing patients to take control of their own care and engage with the app as frequently as they like. These optimizations are likely to improve long-term outcomes in terms of better quality of life, less pain, better mobility, and reduced complications. It should be noted that the conclusions drawn from this article are limited by its nature as a narrative review. The present study did not follow PRISMA reporting guidelines, and therefore has a higher risk of bias than a structured narrative review. The quality of studies included in this review was high, with many implementing randomized allocation to intervention groups.

mHealth apps are still in their infancy, and further research into this growing field is necessary. There is no doubt that this technology will integrate further into all fields of medicine, and it is exciting to witness this significant advancement in patient communication and care.

## Author Contributions

The first draft of the manuscript was written by MB. All authors listed have made a substantial, direct and intellectual contribution to the work, and approved it for publication.

## Conflict of Interest

The authors declare that the research was conducted in the absence of any commercial or financial relationships that could be construed as a potential conflict of interest.
